# Identification of hub genes predicting sensitivity to neoadjuvant chemoradiation in locally advanced rectal cancer

**DOI:** 10.2478/raon-2025-0005

**Published:** 2025-08-06

**Authors:** Qunye Zhao, Chuang Zhang, Xiaotain Zhang, Yanlong Liu, Binbin Cui

**Affiliations:** Department of Colorectal Surgery, Harbin Medical University Cancer Hospital, Harbin, Heilongjiang, China; Center for Disease Control and Prevention, Harbin, Heilongjiang, China; Department of General surgery, The First People’s Hospital of Kunshan, Kunshan, China

**Keywords:** locally advanced rectal cancer, neoadjuvant chemoradiation sensitivity, hub genes, biomarker

## Abstract

**Background:**

Preoperative neoadjuvant chemoradiation (NACR) benefits disease control in most locally advanced rectal cancer (LARC) patients. However, effective biomarkers predicting response to NACR are still not accessible. This study aimed to find potential biomarkers to assess therapy response and susceptibility to LARC.

**Materials and methods:**

Differentially expressed genes (DEGs) between NACR-sensitive and resistant patients were screened using GEO database. STRING and Cytoscape were utilized to construct PPI networks and identify hub genes. Based on CIBERSORT, TCGA, GTEx, GSEA and ROC curves, the connections between hub genes and specific signaling pathways, immune cell infiltration, prognosis value and miRNA-transcription factor (TF)-target network were investigated. Human Protein Atlas (HPA) database was used to visualize hub gene expression in clinical samples.

**Results:**

We identified 2619 up- and 2466 down-regulated genes between NACR-sensitive and resistant patients. The up-regulated DEGs were searched for highly expressed genes in the NACR-resistant, TCGA and GTEx-related datasets compared to the NACR-sensitive group, yielding six hub genes (*RRM2, HNRNPL, EZH2, METTL1, NHP2L1 and ASF1B*). ROC curves demonstrated the predictive utility of the six genes in NACR sensitivity. Immune infiltration research revealed no significant relationship between NACR sensitivity and immune cell infiltration extent. The miRNA-TF-target network of hub genes was established. Finally, HPA database results showed that six genes were expressed at variable levels in rectal cancer patients.

**Conclusions:**

This study identified six hub genes (*RRM2, HNRNPL, EZH2, METTL1, NHP2L1 and ASF1B*) up-regulated in LARC and valuable for predicting patient susceptibility and response to NACR.

## Introduction

Colorectal cancer (CRC) is a serious global health issue, accounting for approximately 1.9 million new cases and 1 million deaths annually. Despite advances in surgical and oncological therapy, it still has a high morbidity and fatality rate. Rectal adenocarcinoma (READ) accounts for about one-third of all cases.^1,2^ Preoperative neoadjuvant chemoradiation (NACR) followed by total mesorectal excision (TME) has become a commonly utilized standard of therapy in clinical practice for patients with locally advanced rectal cancer (LARC) with T3-4, negative/positive lymph nodes, and no distant metastases. The local-regional recurrence rate of LARC can be lowered to 5-9% after multimodal comprehensive therapeutic treatment.^3,4^ Several pathological characteristics, molecular markers including epigenetic, protein and metabolic biomarkers, tumor immuno-microenvironment and non-coding RNA have been investigated for the purpose of finding sensitive, specific as well as accurate markers for the prediction of LARC response following NACR treatment.^5,6^ Most biomarkers, unfortunately, lack sensitivity and specificity. The lack of validation of these findings could be attributed to factors such as patient selection, therapy and technology, tumor heterogeneity, limited sample numbers, and, most critically, varied classifications used to characterize tumor responses.

Immunotherapy is expected to revolutionize LARC treatment in the next years. NACR amplifies the effects of immune-checkpoint inhibitors through the enhancement of CD8+ T-cell infiltration into tumor, the improvement of tumor recognition by host immune system, the increase of tumor elimination by antigen-presenting cells, as well as the remodeling of tumor immune-microenvironment.^7^ Neoadjuvant immunotherapy has recently been demonstrated to be superior in a subset of patients with locally progressing and metastatic colorectal cancer whose tumors contain a deficient mismatch repair protein system (dMMR)^8,9^, the accumulation of mutations in the dMMR results in an excess of neoantigens that stimulate anti-tumor immune responses. In LARC, NACR combined with immunotherapy as a neoadjuvant treatment regimen result in a greater pCR.^10,11^ Thus, successive immunotherapy after NACR treatment could turn immunologically cold tumors into immunologically hot tumors by boosting anti-tumor immunity. Furthermore, research is being conducted to determine if tumors with functionally intact mismatch repair (pMMR) can be treated with immunotherapy.

To summarize, screening individuals with high sensitivity to preoperative NACR as well as LARC patients with high resistance to preoperative NACR for better individualization in clinical practice before treatment is required. Hub genes are a type of gene that is important in numerous cellular physiological functions. A limited number of hub genes in any pathway frequently influence the expression of other genes. We aimed to investigate the relationship and potential mechanisms of differentially expressed genes (DEGs) as well as key genes with tumor immune infiltration in non-responders and responders of LARC patients undergoing NACR in this study, thereby screening specific biomarkers for predicting the sensitivity and efficacy of NACR, which might inform clinical practice work, and improve the prognosis of LARC patients treated with NACR.

## Material and methods

### Datasets and acquisition

READ mRNA expression data for this study were taken through Gene Expression Omnibus (GEO) database.^12^ Three expression profile datasets (GSE119409, GSE123390, GSE150082) were downloaded for analysis. GSE119409 dataset contains READ pre-therapy biopsy samples from 66 cases, of which 41 were radiotherapy-resistant, 15 were radiotherapy-sensitive, and 10 were unknown; GSE123390 dataset contains 28 rectal cancer samples, of which 17 were NACR-resistant, 11 were NACR-sensitive, and 5 normal rectal tissue; GSE150082 dataset contains READ preoperative biopsy samples from 39 cases, 23 of which were NACR-resistant and 16 of which were NACR-sensitive. Selected DEGs with NACR resistance and sensitivity were separated into two groups for comparative investigation.

Genotype-Tissue Expression (GTEx, [http://ge-nome.ucsc.edu/gtex.html]) provided gene expression data for normal human tissues.^13^ The gene expression data of rectal cancer tumor tissues were obtained from The Cancer Genome Atlas database (TCGA, [https://portal.gdc.cancer.gov/]), a public database established by the National Institutes of Health and the Cancer Institute, which integrates genetic data, clinical data and image data of various tumor types. The RNA-Seq data of rectal cancer tissue were obtained from TCGA database, including 92 tumor samples. Data of normal tissue were obtained from TCGA and GTEx databases, including 10 and 308 normal samples respectively. All data are available online for free.

### Identification of differentially expressed genes

This work used the R program “preprocessCore”^14^ and the “limma”^15^ package (R Studio 4.2.1) to detect and filter DEGs. The three datasets were subjected to data homogenization, gene symbol annotation, and analysis of variance. P < 0.05, Log2FC>|0| were used to test for differential genes. And volcano plots were draw by R packages “limma” and “ggplot2”.^16^

### Functional enrichment analysis

DEG GO, KEGG as well as GSEA were undertaken using “clusterProfiler”^17^ as well as “enrichplot” packages^18^ in R. Immunologic signature gene sets, from the Molecular Signatures Database (MsigDB), were utilized as a reference for GSEA.

### Visualization of gene networks

Protein-protein interaction (PPI) networks were built for differential genes utilizing STRING database (https://cn.string-db.org/)^19^ as well as visualized utilizing Cytoscape software (version 3.9.1).^20^

### Hub gene identification

The top 20 genes in PPI networks were found using the CytoHubba plugin (Betweenness algorithm) in Cytoscape software.^21^ The following parameters must be met in order to screen Hub genes among the top 20 genes. (1) Highly expressed in the NACR resistance group; (2) Highly expressed in TCGA rectal cancers (READ) in tumor tissues compared to TCGA normal tissues combined with GTEx normal tissues; (3) Highly expressed in paired tumor tissues and paracancerous tissues in the TCGA database. Box plots were created using the R software tools “ggplot2” as well as “ggpubr” (R Studio 4.2.1) to compare the expression levels of the critical genes in CRC and normal samples. The “circlize” package^22^ in R was used to perform correlation analysis of six key genes on the three datasets. The “pROC” package^23^ in R was utilized for visualization of ROCs of the six genes in the three datasets for the prediction of patient resistance. Using GSE150082 data, a correlation study of the six genes with all genes was also done.

### Immunocyte infiltration analysis

For the three data sets, the degree of immune cell infiltration was determined using “CIBERSORT” package in R^24^, with the expression levels of the various immune cells displayed as bar graphs. The percentage of immune cell infiltration was compared between the NACR-sensitive and resistant groups, and Spearman’s correlation was calculated and visualized using the “ggplot2” package (R Studio 4.2.1). Statistical significance was defined as p < 0.05.

### Validation of the protein expression levels of the hub genes

The UALCAN database was used to explore the proteomic profiling of six hub genes in colorectal cancer tissues and normal tissues.^25^ We also downloaded immunohistochemistry (IHC) information via the Human Protein Atlas (HPA) database (https://www.proteinatlas.org/)^26^ to further confirm protein expression levels of the six hub genes in colorectal cancer and normal tissues. The HPA can provide IHC data based on proteomics for a large variety of proteins in malignant and normal tissues.

## Results

### DEGs screening for NACR-sensitive and resistant samples

**FIGURE 1.**
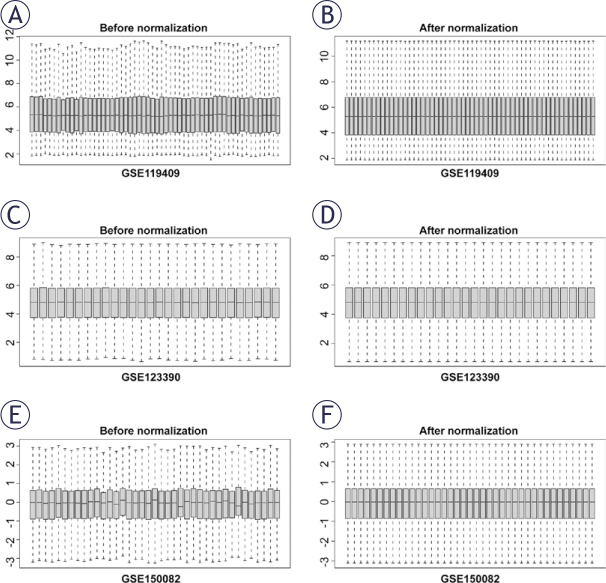
Homogenization of the three datasets. The preprocessCore package was used for data normalization of **(A-B)** GSE119409, **(C-D)** GSE123390, and **(E-F)** GSE150082 datasets.

**FIGURE 2.**
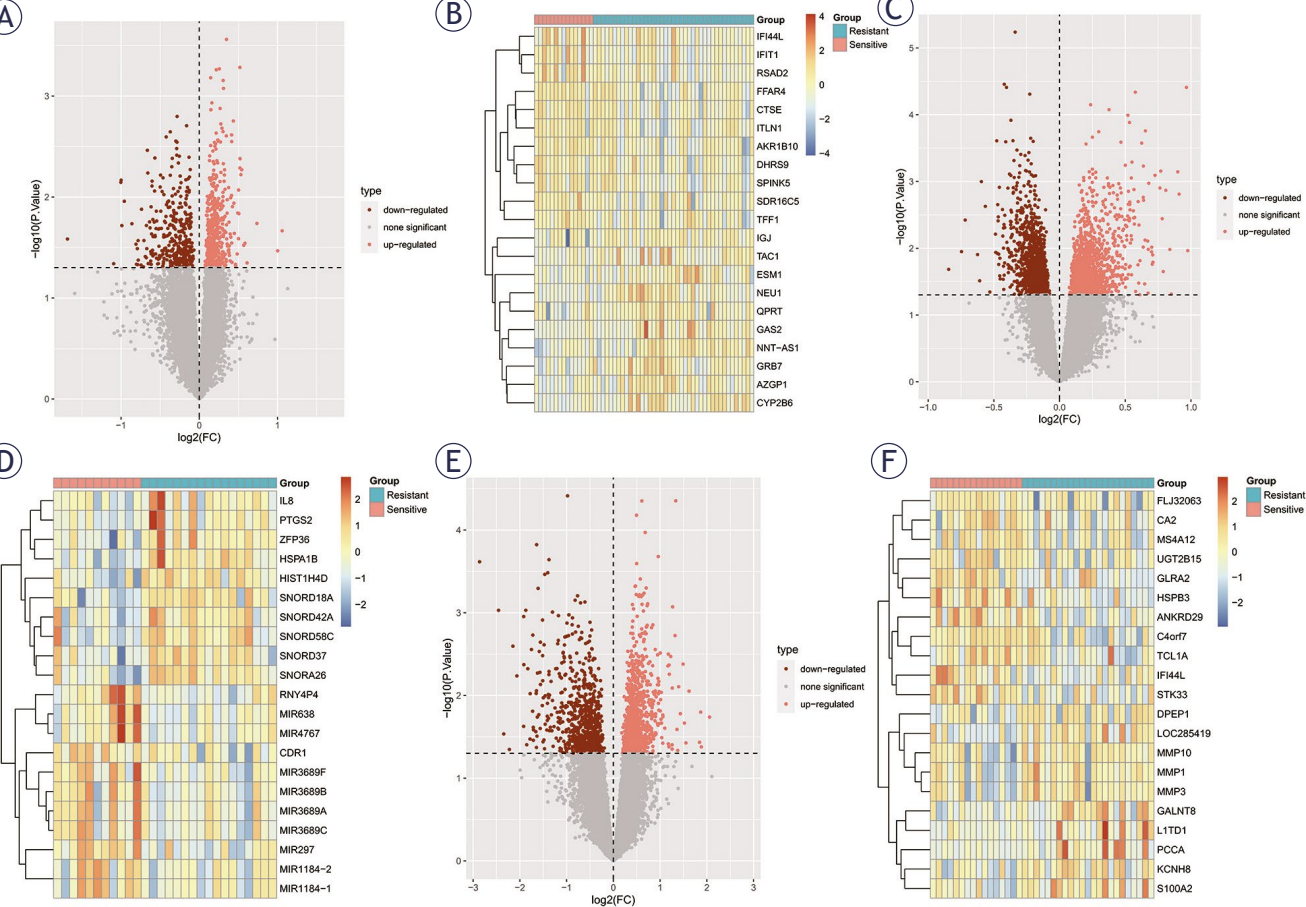
Differentially expressed genes (DEGs) volcano plot as well as heatmap. **(A, C, E)** DEGs volcano plot in GSE119409, GSE123390, and GSE150082 datasets, respectively. Pink: up-regulated; Red: down-regulated. **(B, D, F)** DEGs heatmap between resistant (blue) and sensitive (pink) of locally advanced rectal cancer (LARC) to neoadjuvant chemoradiation (NACR). Blue: low expression level; Red:high expression level.

The samples from the three datasets were classified into NACR-sensitive and NACR-resistant groups based on the three datasets (Figure 1) adjusted for homogenization by “preprocessCore” package, with DEGs between the sensitive and resistant groups screened using the criteria of P < 0.05, Log2FC>|0|. Differential expression analysis was used to screen 512 up- and 349 down-regulated genes in GSE119409; 1171 up- and 1355 down-regulated genes in GSE123390; and 936 up- and 762 down-regulated genes in GSE150082. And heat maps were displayed on the volcano (Figure 2).

### Functional enrichment of DEGs

**FIGURE 3.**
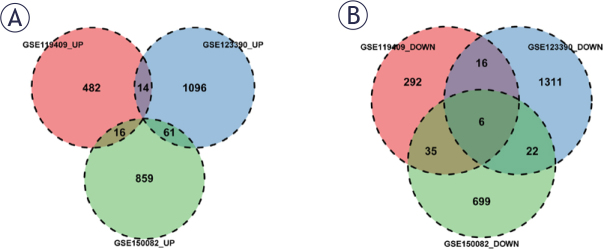
Exploration of differentially expressed genes between neoadjuvant chemoradiation (NACR)-resistant and NACR-sensitive rectal adenocarcinoma (READ) samples based on GSE119409, GSE123390, GSE150082 datasets. **(A)** The up-regulated gene intersections among three datasets. **(B)** The down-regulation gene intersection among three datasets.

The DEGs identified in each of the three datasets were intersected, and genes that were either concurrently up-regulated or concurrently down-regulated in at least two of the datasets were chosen for further investigation. The up-regulated DEGs had 91 concurrently up-regulated genes and the down-regulated DEGs contained 79 concurrently down-regulated genes, of which 6 were concurrently down-regulated in all three datasets (Figure 3).

**FIGURE 4.**
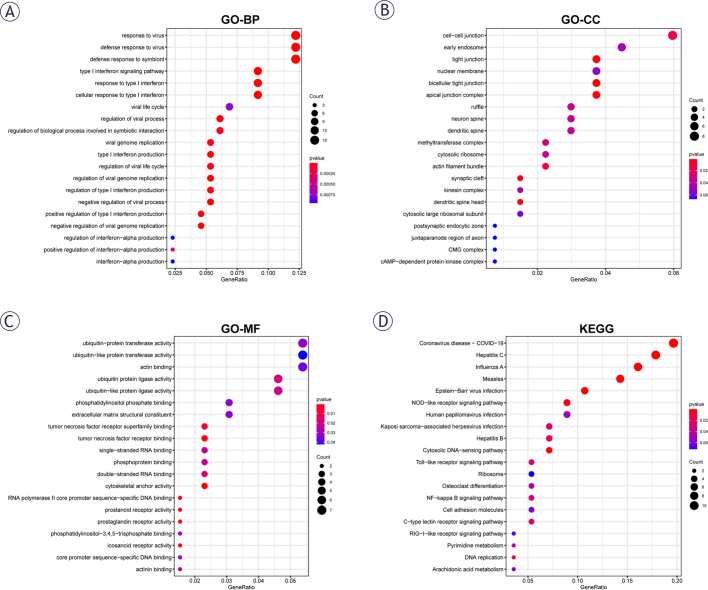
Gene ontology (GO) and Kyoto Encyclopedia of Genes and Genomes pathways (KEGG) enrichment analysis of up- and downregulated genes. **(A-C)** GO function enrichment analysis including biological processes (BP), cellular component (CC), and molecular function (MF). **(D)** KEGG term analysis of differentially expressed genes (DEGs).

For further exploration of the underlying DEGs biological functions and signaling pathways, GO as well as KEGG analyses were undertaken utilizing ‘clusterprofiler’ package. This indicated that the most enriched GO terms among biological process (BP), cellular component (CC) as well as molecular function (MF) included response to virus, cell-cell junction as well as ubiquitin-protein transferase activity, respectively (Figure 4A–C). In addition, DEGs mainly enriched in pathways involving virus infection-related, immune-related as well as inflammation-related pathways, such as NOD-like receptor signaling pathway, Toll-like receptor signaling pathway, NF-kappa B signaling pathway. Besides, various DEGs were also involved in pathways such as pyrimidine metabolism, DNA replication, arachidonic acid metabolism (Figure 4D).

### Identification of hub genes from DEGs

**FIGURE 5.**
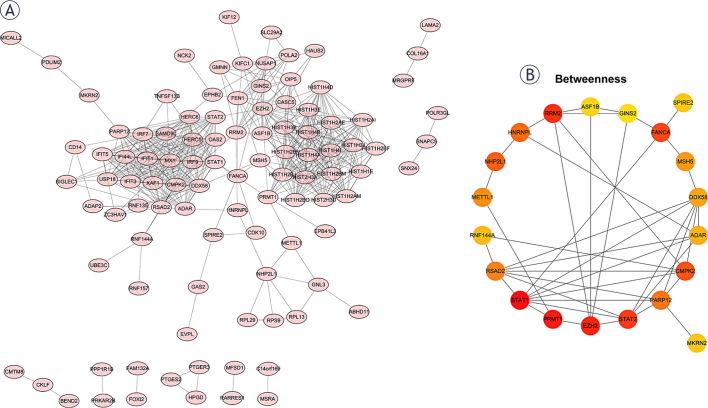
Protein interaction network construction (PPI) network. **(A)** PPI for differential genes using string database, visualized using Cytoscape software. **(B)** The Betweenness algorithm of cytoHubba plug-in of cytoscape software was used to identify the top 20 hub genes in the PPI network.

**FIGURE 6.**
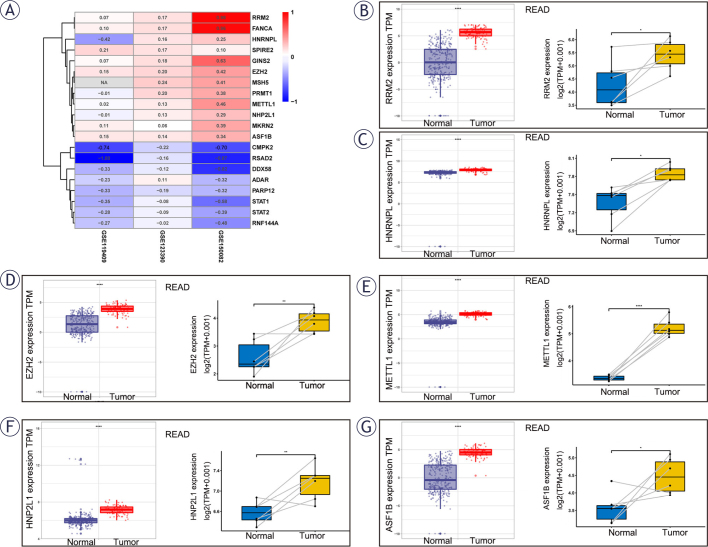
Hub gene validation. **(A)** Heatmap of logFC values of top 20 genes in the GSE119409, GSE123390, GSE150082. **(B-G)** 6 hub genes validation at gene expression level in rectal adenocarcinoma (READ) tissues and normal tissues using The Cancer Genome Atlas database (TCGA) and Genotype-Tissue Expression (GTEx) databases.

PPI networks were created for differential genes utilizing STRING database and visualized utilizing Cytoscape software. The top 20 core genes in PPI network were then identified using the Cytoscape software Betweenness algorithm of the cytoHubba plugin (Figure 5). The magnitude of log2FC values of the 20 genes in the three datasets was then analyzed and presented as a heatmap (Figure 6A) (numerical values indicate logFC values, greater than 0 indicates up-regulation of expression in the drug-resistance group, and NA indicates that the MSH5 gene is missing from GSE119409).

The hub genes were then chosen from among the top 20 genes that met the following criteria: (1) the genes were highly expressed in the NACR-resistant group; (2) the genes were highly expressed in tumor tissues in the TCGA-READ database compared to normal tissues in the TCGA database combined with the normal tissues in the GTEx database (left panel in the box), which were visualized by the R software “ggplot2” package; (3) genes were highly expressed in paired tumor tissues and paracancer tissues The goal of this hub gene screening was to find common targets for tumor and medication resistance, i.e., genes that may be targeted to both decrease tumors and boost NACR sensitivity. A total of 6 hub genes were screened: RRM2, HNRNPL, EZH2, METTL1, NHP2L1, ASF1B (Figure 6B–G).

**FIGURE 7.**
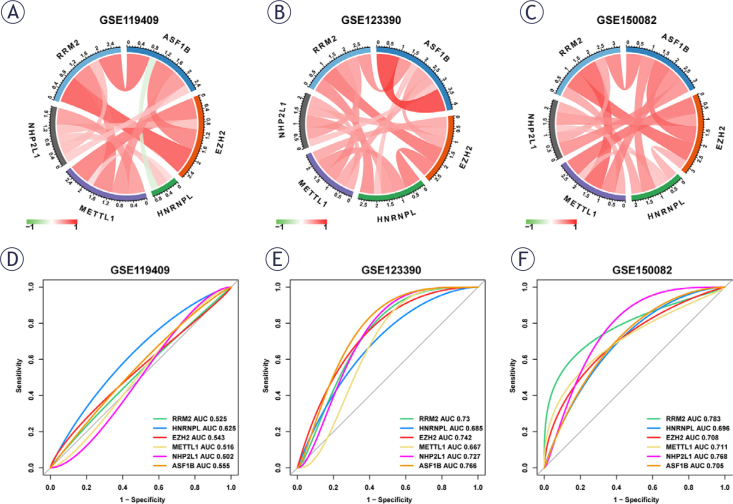
Hub gene validation for diagnostic value. **(A-C)** Correlation analysis of 6 hub genes in GSE119409, GSE123390 as well as GSE150082. **(D-F)** ROC curves of the 6 hub genes for the prediction of neoadjuvant chemoradiation (NACR) resistance in patients in GSE119409, GSE123390 and GSE150082.

Three datasets were employed in the ROC analysis for correlation analysis of the six core genes. The correlation was visualized via “circlize” package, with positive correlations displayed as red lines and negative correlations as green lines; the darker the color, the stronger the correlation (Figure 7A–C). The ROC curves and AUC statistics of the six genes in the three datasets were then generated using the R software “pROC” package for patient resistance prediction for the evaluation of CRC diagnosis sensitivity and specificity (Figure 7D). AUC values greater than 0.7 for numerous hub genes were highly predictive of NACR sensitivity. ASFIB had an AUC value of 0.766 in the GSE123390 dataset and an AUC value of 0.783 in the GSE150082 dataset. These findings demonstrated that six hub genes displayed superior predictive value of NACR in LACR.

### Immune Infiltration in LARC patients receiving NACR treatment

For the aim of exploring the specific cell types that caused differences in immune infiltration between NACR-sensitive group and NACR-resistant group, we used “CIBERSORT” to estimate the immune cell types abundance of the samples, and visualized using the R package “ggplot2”. Figure 8A, C, and E depicts the percentage of immune cell infiltration per sample. We found high infiltrating levels of T cells, NK cells and macrophages in three datasets. The difference in immune cell infiltration between the resistant and sensitive groups is shown in Figure 8B, D, and F. The results demonstrated that there were significant differences only in monocytes (P = 0.037) in CSE123390, B-cell naivety (P = 0.016), and mast cell activation (P = 0.007) in GSE150082 between NACR-sensitive and NACR-resistant tissues, and other immune cell infiltration was not substantially associated to NACR sensitivity in rectal cancer (P > 0.05).

**FIGURE 8.**
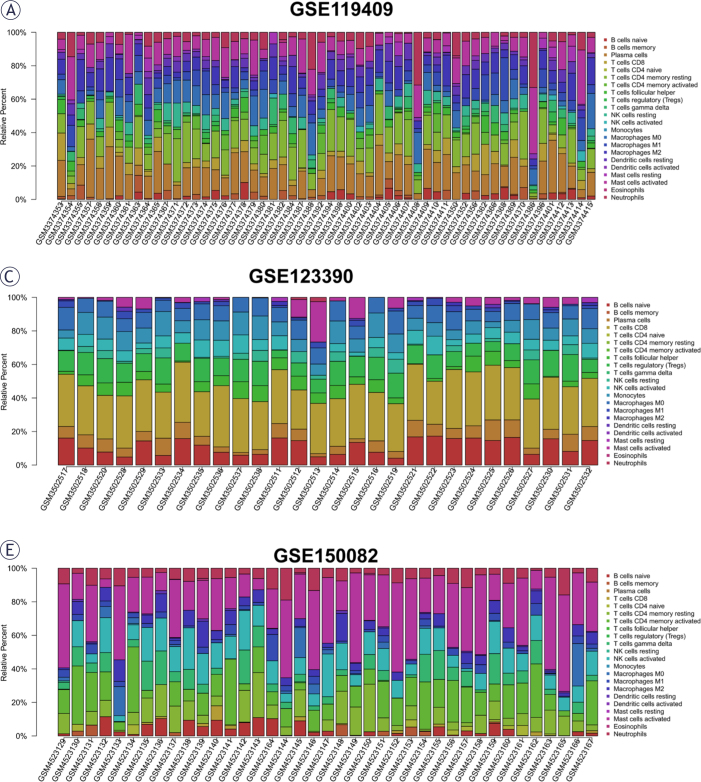
Assessment of the degree of immune cell infiltration in GSE119409, GSE123390 and GSE150082 datasets. **(A, C, E)** showing the proportion of immune cell infiltration per dataset.

**FIGURE 8.**
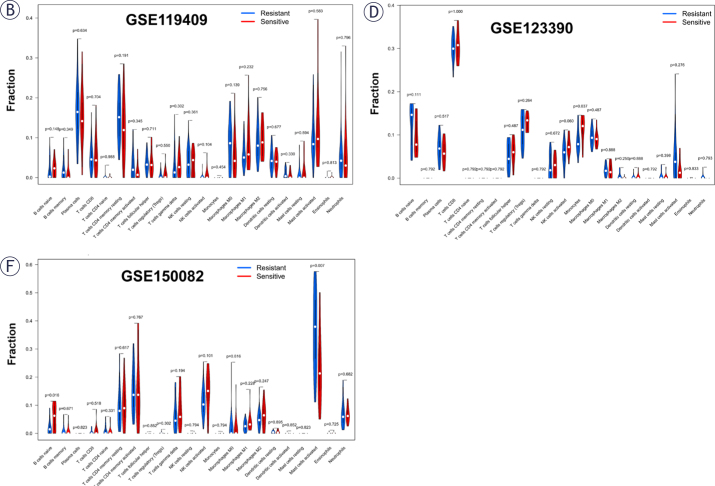
**(B, D, F)** showing the difference in immune cell infiltration between the resistant and sensitive groups.

### Enrichment analysis of GSEA gene sets

6-hub-gene correlation analysis with all genes was conducted using GSE150082, investigating the key biological activities and significant pooled signaling pathways of hub genes, and we used heatmaps to display the top 50 significantly linked genes (Supplementary Figure 1). Then, using the correlation analysis results in Supplementary Figure 1, we performed single-gene Reactome-based GSEA of 6 hub genes using the R software “clusterprofiler” package. The top 20 findings for each of the six genes are given separately (Supplementary Figure 2). Greater than 0 Numerical Expression Enrichment Score (ES) values indicate that the gene is positively correlated with the pathway and vice versa. ASF1B and EZH2 were mainly enriched in Cell Cycle, and Cell Cycle Checkpoints pathways; HNRNPL was mainly enriched in Cell Cycle, Metabolism of RNA, rRNA processing and transcriptional gene expression regulation pathways; METTL1 is mainly enriched in transcriptional gene expression regulation, Pyruvate metabolism and Citric Acid (TCA) cycle, Death Receptor Signaling pathway and oncogenic pathways; NHP2L1 is mainly enriched in translation and RNA metabolism, rRNA processing, and cell cycle signalings. RRM2 was associated with the RNA metabolism, DNA replication, cell cycle process, and generic transcription pathways (Supplementary Figure 2). Most hub gene were correlated with cell cycle process and transcriptional regulation of gene expression, suggesting their potential mechanism in READ progression. Finally, the Regnetwork database identified miRNAs and transcription factors upstream of the six hub genes, which were visualized using Cytoscape software (Supplementary Figure 3).

### Exploration and validation of the expression levels of 6 hub genes in clinical samples

Based on the UALCAN database, the protein expression and/or phosphorylation level of 6 hub genes in colorectal cancer was explored. As shown in Supplementary Figure 4, the protein expression of most hub genes and/or phosphorylation level was significantly upregulated in colorectal cancer, while the phosphorylation level of ASF1B showed no significance difference between the normal and tumor samples.

Furthermore, we explored the protein expression of hub genes in READ using HPA database. RRM2 expression in normal rectal tissue is moderately high. Since no information about RRM2 expression of READ was available in HPA database, we instead discovered RRM2 expression in colon cancer. RRM2 expression levels in colon cancer patients’ tumor tissues ranged from moderate to low to no detectable protein expression. HNRNPL was strongly expressed in rectal normal tissues, with nuclear HNRNPL staining also seen in READ tissues (Supplementary Figure 5). EZH2 was found in high concentrations in normal rectal tissues and in varying high and medium concentrations in rectal cancer tissues. METTL1 expression was moderate in normal rectal tissues and high in rectal cancer tissues. NHP2L1 expression was low in normal rectal tissues. NHP2L1 was expressed at varying levels in rectal cancer patients’ tumor tissues, ranging from medium to low to undetectable protein expression. NHP2L1 expression was low in normal rectal tissues and moderate in rectal cancer tissues. METTL1 expression was low in normal rectal tissues and moderate in rectal cancer tissues. ASF1B was expressed at low levels in normal rectal tissues and at medium levels in cancerous rectal tissues. Individual variations in NACR sensitivity and inherent biological disparities in biological parameters of rectal cancer patients may be influenced by the variable expression levels of hub genes.

## Discussion

Rectal cancer patients who utilize NACR have a better likelihood of achieving complete pathological remission. A thorough understanding of the mechanisms driving NACR sensitivity is critical for enhancing LARC efficacy. However, in ordinary clinical practice, molecular techniques for early identification or screening of CRC for NACR sensitivity and efficacy remain scarce.^27^ As a result, we used bioinformatics to investigate the biomarkers as well as putative mechanisms underlying NACR resistance in rectal cancer.

Some previous studies have also explored the genomic patterns related to therapeutic response of rectal cancer patients based on bioinformatics analysis. For example, Zhao *et al*. have investigated the biomarkers related to the sensitivity of radiotherapy in READ and identified three hub genes, including PLAGL2, ZNF337 and ALG10.^28^ Afshar *et al*. have reported four hub genes including ZEB2, JAM2, NDN, and PPAP2A were associated with the response of colorectal tumors to chemoradiotherapy based on Weighted Gene Co-Expression Network Analysis and CytoHubba plugin.^29^ Marinkovic *et al*. have shown that tumor morphology and hematological parameters were of clinical significance to predict the favorable response to NACR via the logistic regression model.^30^ The present work identified DEGs between NACR resistant and sensitive patients utilizing GSE119409, GSE123390 as well as GSE150082. We interested the DEGs in these three datasets, and a total of 2619 up- as well as 2466 down-regulated were identified, which were markedly enriched in various cancer biological processes. Finally, 6 novel hub genes (*RRM2, HNRNPL, EZH2, METTL1, NHP2L1 and ASF1B*) are found highly expressed in READ and regarded as hub genes.

Furthermore, six hub genes were found to be in correlation with various disease-related gene expression levels, such as TP53, MKI67 as well as POLE. GSEA analysis results revealed RRM2, HNRNPL, EZH2, METTL1, NHP2L1 and ASF1B might involve multiple disease progression signaling pathways. For instance, ASF1B and EZH2 implicated Cell Cycle, Mitotic and Cell Cycle Checkpoints signaling pathways. METTL1 affected signaling downstream of RAS mutants, Paradoxical activation of RAF signaling by kinase inactive BRAF, signaling by RAS mutants, signaling by moderate kinase activity BRAF mutants. The ROC curves and AUC statistics for patient resistance prediction based on varying expression of RRM2, HNRNPL, EZH2, METTL1, NHP2L1, and ASF1B in the three datasets revealed that hub genes have a strong predictive value for NACR susceptibility, suggesting their potential value in risk stratification and improving the therapeutic therapies.

Finally, regulatory network showed that RRM2, HNRNPL, EZH2, METTL1, NHP2L1 and ASF1B were regulated by various TFs. Moreover, six hub gene expression levels in clinical samples were further validated through HPA database, demonstrating that they in READ patients varied strongly. This might reveal NACR expression difference among various individuals.

RRM2, called ribonucleotide reductase regulatory subunit M2. RRM2 confers the enzymatic activity of the ribonucleotide reductase complex, which represents the rate-limiting enzyme in DNA synthesis and as such is required for DNA replication and DNA repair ^31^. RRM2 has been reported as a cancer driver as well as an oncogenic target, and its overexpression is also widely and critically expressed in various chemo-resistant tumors.^32^ Studies showed strong and ubiquitous RRM2 mRNA and protein expression in chemoresistance. RRM2 was discovered to be linked to DOX resistance in DOX-resistant breast cancer microarray.^33^ For the selection of chemotherapy responders, RRM2 might serve as a valuable tumor biomarker. Elevated RRM2 mRNA expression was linked to poor chemosensitivity as well as adverse prognosis.^34^ Moreover, the upregulation of RRM2 also induces chemo-resistance to cisplatin and fluorouracil (5-FU) via activating epidermal growth factor receptor (EGFR)/AKT proliferative pathway.^35^ RRM2 elevation impacts intricate pathways for the mediation of tumor survival, growth, apoptosis as well as chemoresistance. Hence, RRM2 might emerge as a candidate for overcoming NACR resistance, and RRM2 expression or activity inhibition might represent a valuable NACR strategy through enhancement of cell apoptosis, suppression of cell proliferation, and interference with DNA replication and repair. Few studies have reported the impact of RRM2 in neoadjuvant chemoradiotherapy in rectal cancer.

HNRNPL, named as heterogeneous nuclear ribonucleoprotein L. As a member of the hnRNP family, it exhibits an essential function in the occurrence and development of bladder cancer, gastric cancer as well as other cancers.^36,37^ Wang *et al*.^38^ reported that HNRNPL was elevated in CRC, and HNRNPL reduction suppressed cell growth as well as metastasis. Meanwhile, HNRNPL knockdown induced CRC cells to apoptosis. Another study showed that HNRNPL overexpression enhanced tumor growth in vivo.^39^ These findings are in agreement with our present work. HNRNPL is highly expressed in both rectal cancer tumor tissue and NACR resistance group, suggesting that reducing the expression of HNRNPL in LACR may increase the sensitivity of patients to NACR, thus achieving a better prognosis.

EZH2, named as enhancer of zeste 2 polycomb repressive complex 2 subunit. The EZH2 gene is found on human chromosome 7q35 and spans almost 40 kb, with 20 exons and 19 introns, with exon lengths of 41 323 bp and intron lengths of 0. 15 17.7 kb. The Ezh2 gene is involved in cell proliferation, differentiation, and tumor development.^40^ Several investigations have demonstrated a connection between EZH2 and RUNX3 silencing in rectal cancer.^41,42^ RUNX3 expression has been demonstrated to be regulated by EZH2.^43^ Methylation is a significant contributor to the poor expression of the RUNX3 gene in a variety of malignant tumors.^44^ According to one study, EZH2 can cause epigenetic silencing in glioma cells by causing lysine methylation at the 27th position of RUNX3’s H3 histone protein.^45^ Another study found that when LACR patients were given neoadjuvant chemotherapy, those with low EZH2 expression had a better tumor regression response and a better prognosis than those with high EZH2 expression. Patients with high expression of RUNX3 showed better tumor regression response and down-staging compared with those with low expression of RUNX3.^46^ There is currently few research on EZH2 expression alterations before and after rectal cancer neoadjuvant therapy. Our findings reveal that EZH2 is substantially expressed in rectal cancer tissues, which is consistent with the findings of other solid tumor studies. Preoperative identification of EZH2 protein expression in tumor tissues in patients with locally advanced rectal cancer can screen out the group with a higher likelihood of benefit from neoadjuvant chemotherapy. Alternatively, the safety and efficacy of combining EZH2 targeting medications to improve NACR sensitivity while patients get neoadjuvant therapy can be investigated further, assisting physicians in making treatment decisions for corresponding patients and benefiting patients.

METTL1, named as methyltransferase-like 1, is located on a region of chromosome 12 (12 q13-14) that is frequently amplified in cancers. It mediates N(7)-methylguanine (m7G) formation as well as regulates mRNA translation.^47^ Abnormal METTL1 level was tightly linked to tumorigenesis as well as cancer progression in previous studies.^48,49^ Upregulation of METTL1 expression has recently been found to increase oncogenic activity and alter anticancer treatment resistance. In one study, METTL1 overexpression increased the lethal impact of cisplatin via the S100A4/p53 pathway on cisplatin-resistant colon cancer cells.^50^ Another study discovered that METTL1 is frequently amplified and over expressed in malignancies, which is linked to poor patient survival. METTL1 deficiency reduces the quantity of m7G-modified tRNAs, alters the cell cycle, and decreases oncogenicity. METTL1 over expression, on the other hand, promotes oncogenic cell transformation and cancer.^51^ We discovered that METTL1 was substantially expressed in tumor tissues and NACR resistance groups in our investigation by screening differential genes in patients with variable responses to NACR, and IHC results in the HPA database validated this conclusion. This finding is essentially similar with the previous findings; hence this work highlights the potential of METTL1 as an anticancer therapeutic target to boost NACR sensitivity.

ASF1B, named as anti-silencing function 1B histone chaperone. Anti-silence function 1 (ASF1), a histone H3-H4 chaperone protein, is involved in DNA replication, DNA damage repair, and transcriptional control. ASF1 in mammals is made up of two homologous proteins, ASF1A and ASF1B.^52^ Overexpression of ASF1B has been associated with tumor growth and metastasis in various malignancies, including lung adenocarcinoma^53^, cervical cancer^54^, hepatocellular carcinoma^55^, breast cancer^56^, and thyroid carcinoma.^57^ ASF1B is strongly expressed in most tumor tissues relative to normal tissues, according to a pan-cancer investigation, and there is a link between ASF1B expression and clinical prognosis. Furthermore, ASF1B expression is associated with poor prognosis in main tumor types and may be an independent prognostic factor in many malignancies. A study has also reported that ASF1B is positively correlated with the TNM stage of colorectal cancer patients. Silencing of ASF1B significantly represses colorectal cancer cell proliferation, migration, invasion, stemness and epithelial mesenchymal transition progression via the PI3K/AKT pathway.^58^ However, the precise involvement of ASF1B in each tumor type needs be explored further.^59^ However, the relevance of ASF1B in rectal cancer radiation and chemotherapy resistance has not been thoroughly researched or debated. In this study, we found that ASF1B expression levels were higher in the NACR resistant group when we compared the differences between patients who received varied NACR responses. ASF1B is primarily enriched in Cell Cycle, Mitotic, and Cell Cycle Checkpoints. These findings are consistent with previous research, and ROC curve analysis revealed that ASF1B is a reliable predictor of NACR sensitivity in LARC patients. These findings could assist to explain the role of ASF1B in tumor growth and give novel regulatory targets for more precise and tailored anti-tumor chemoradiotherapy treatments.

This gene was originally known as NHP2L1, but its official symbol is now SNU13, and its full name is small nuclear ribonucleoprotein 13. Enables ATPase binding activity and RNA binding activity. Contributes to snoRNA binding activity. Involved in mRNA splicing, via spliceosome. In one study, SNU13 participated in the development of SF-risk-models to predict the prognosis of breast cancer as one of 12 splicing factors (SFs) genes, and produced good prognosis-predicting findings.^60^ However, the relevance of SNU13 in colorectal tumorigenesis and development remains unknown.

There is evidence that colorectal cancer cells exhibit rich mutational diversity with higher somatic mutation burden compared to normal colorectal cancer stem cells, and single cancer cells have genetic variations. Furthermore, even tightly related cells located within the same cancer site respond differently to anticancer drugs.^61^ The HPA database revealed that hub gene expression varied in individual colorectal tumors in our investigation, which is consistent with the preceding findings. Furthermore, multiple clinical trials are now being conducted to investigate sequential immune checkpoint inhibitors after neoadjuvant radiotherapy as a preoperative neoadjuvant therapeutic option for LACR. Preliminary results reveal that the combined radiation and immunotherapy group had much better tumor regression rates than the traditional NACR method, with pCR rates of up to 48% in all cohorts and up to 60% in the high microsatellite instability cohort.^10,11^ The potential synergistic processes, however, are unknown. We detected no significant link between NACR treatment sensitivity and immune-infiltrating cells in this study, which suggests that the potential mechanism of NACR paired with immunotherapy has to be examined further.

MicroRNA is a short-stranded regulatory ncRNA (regulatory non-coding RNA) that can be induced to express and then regulate the expression of protein-coding genes under particular situations. MiRNA attaches to the target mRNA with relative specificity, that is, it binds to the target mRNA’s 3’-untranslated region (3’-UTR) in a partially complementary fashion, limiting translation or changing its stability, and subsequently contributes in cell proliferation. MiRNAs bind to target mRNAs in a relatively specific manner, i.e., they bind to the 3’-untranslated region (3’-UTR) of target mRNAs in a partially complementary manner to inhibit translation or affect stability, and then participate in biological processes such as cell proliferation, differentiation, apoptosis, and division.^62^ More and more evidence suggest a link between aberrantly expressed miRNAs and tumor radiosensitivity and tolerance. For example, Hu *et al*. discovered that miRNA-214 was highly expressed in radiosensitive colorectal cancer specimens and negatively correlated with the plasma level of CEA, and discovered that miRNA-214 was able to enhance colorectal cancer radiation sensitivity by inhibiting ATG12-mediated autophagy phenomenon.^63^ This study built a network of miRNAs and transcription factors predicting the upstream of the six hub genes to fully understand the sixhub-gene roles as well as potential mechanisms on READ radio-responsiveness as well as prognosis.

This research still has several limits and flaws. For starters, the sample size obtained from the TCGA and GEO datasets was tiny. Second, the results lack in-vivo as well as in-vitro assay validation. Second, the underlying mechanisms of these hub genes in NACR resistance in LARC remain unclear. Notwithstanding these flaws, our early work still provided valuable and informative results. The role of hub genes in NACR sensitivity should be explored using preclinical studies in the future.

In conclusion, six key genes including *RRM2, HNRNPL, EZH2, METTL1, NHP2L1*, and *ASF1B* were identified to predict the therapeutic response to NACR in LARC patients, which might provide novel biomarkers for risk stratification and clinical treatment guidance of LARC patients. Future study is expected to explore the function and underlying mechanism of these key genes involved in READ progression.

## Supplementary Material

Supplementary Material Details
