# Supplementary material for: Identification of hub genes predicting sensitivity to neoadjuvant chemoradiation in locally advanced rectal cancer
Source: Radiol Oncol. 2025 Aug 6;59(3):435–49. doi: 10.2478/raon-2025-0005 (PMC12441892; doi:10.2478/raon-2025-0005)
Supplement: Supplementary file 1 — Supplementary Material Details [file raon-2025-0005_sm.pdf]

Radiology and Oncology | Ljubljana | Slovenia | www.radioloncol.com

Identification of hub genes predicting sensitivity to neoadjuvant chemoradiation in locally advanced rectal cancer

Qunye Zhao, Chuang Zhang, Xiaotain Zhang, Yanlong Liu, Binbin Cui

doi: 10.2478/raon-2025-0005

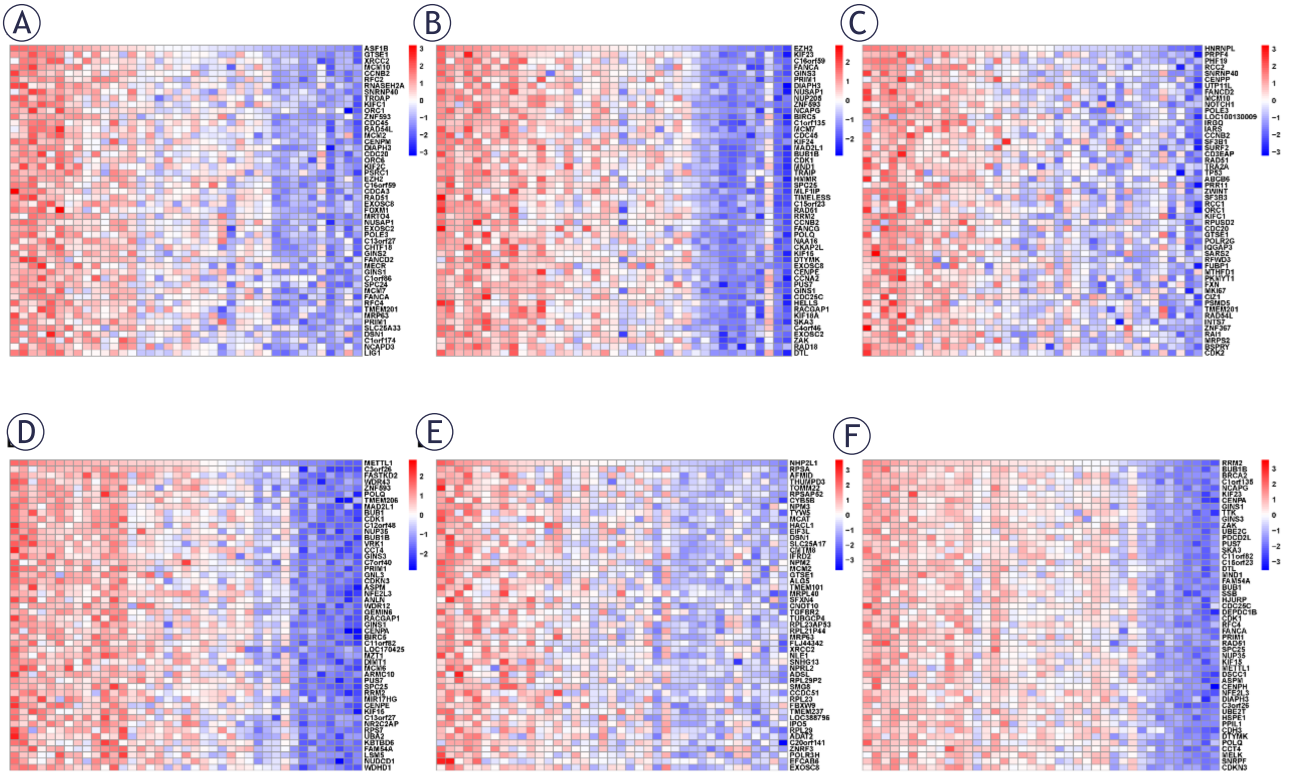

SUPPLEMENTARY FIGURE 1. (A-F) Heatmap of expression correlation between six hub genes and top 50 significantly linked genes in rectal adenocarcinoma (READ).

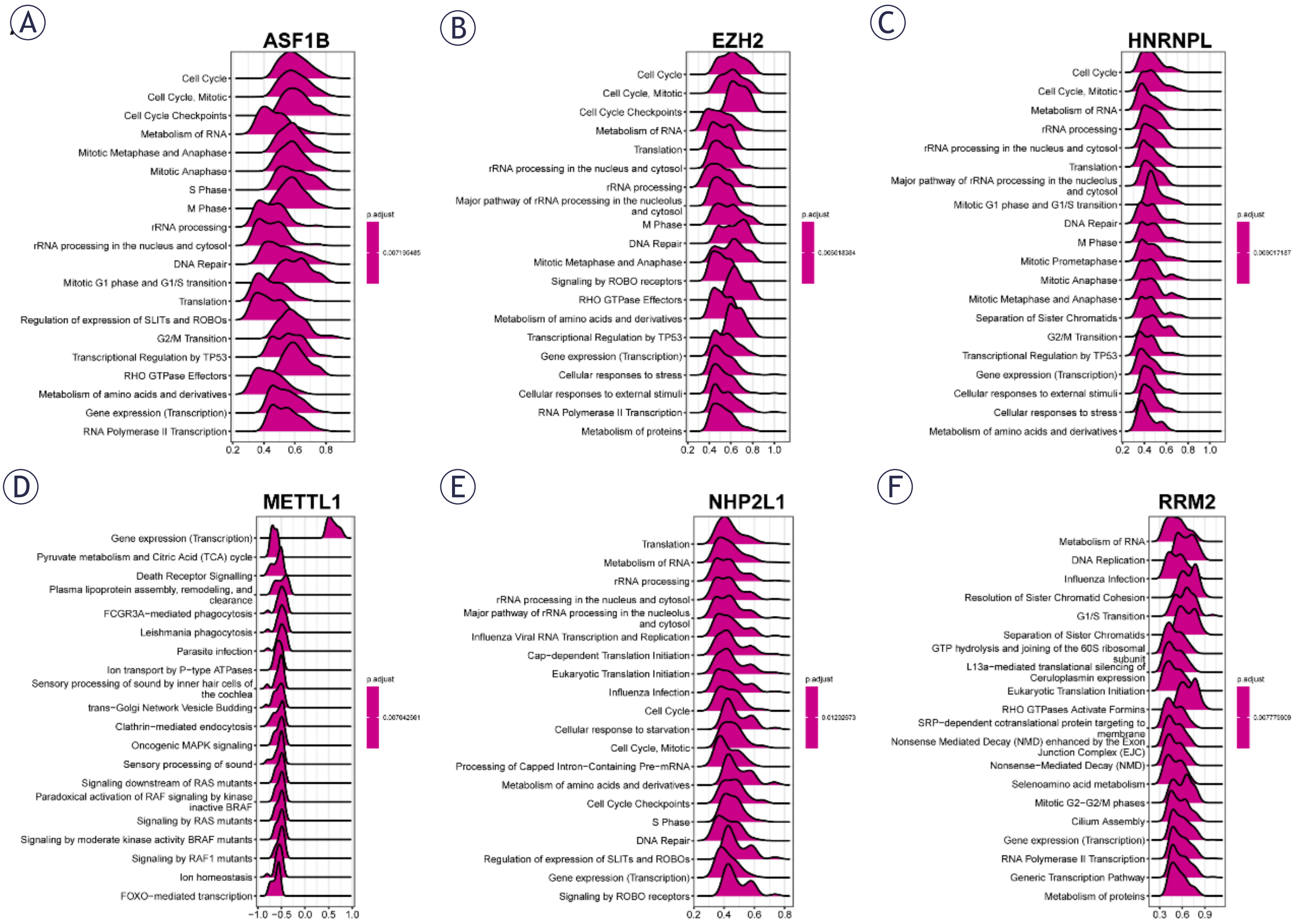

SUPPLEMENTARY FIGURE 2. (A-F) Single-gene Reactome-based GSEA of six hub genes.

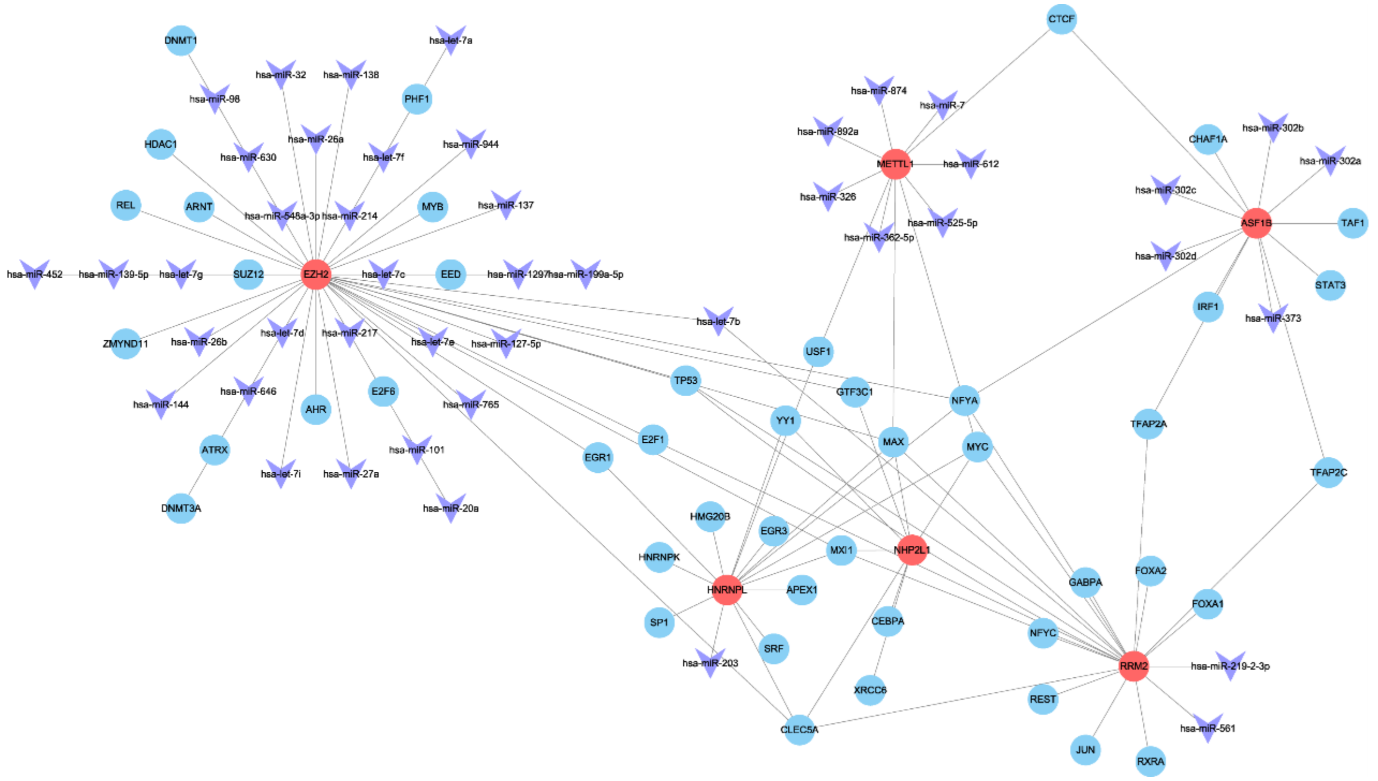

SUPPLEMENTARY FIGURE 3. miRNA-TF-target network analysis of hub genes. The miRNAs and transcription factors of the hub genes were explored using Regnetwork database and visualized using Cytoscape software. Red circles represent hub genes. Blue circles represent TFs of hub genes, and purple V-shape nodes represent miRNAs.

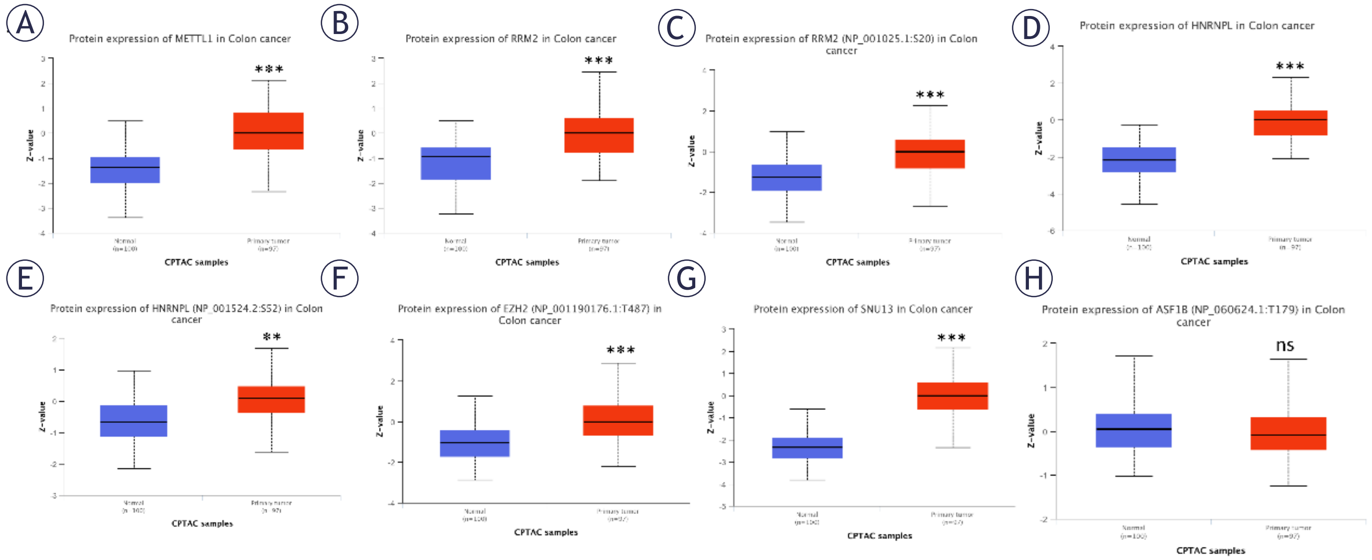

SUPPLEMENTARY FIGURE 4. The protein expression and/or phosphorylation level of RRM2, HNRNPL, EZH2, METTL1, NHP2L1 and ASF1B in colorectal cancer. UALCAN database was used to explore the **(A)** METTL1 protein expression, **(B)** RRM2 protein expression, **(C)** RRM2 phosphorylation level, **(D)** HNRNPL protein expression, **(E)** HNRNPL phosphorylation level, **(F)** EZH2 phosphorylation level, **(G)** SNU13 (NHP2L1) protein expression, and **(H)** ASF1B phosphorylation level in colorectal cancer.

** P < 0.01; *** P < 0.001; ns = non-significant

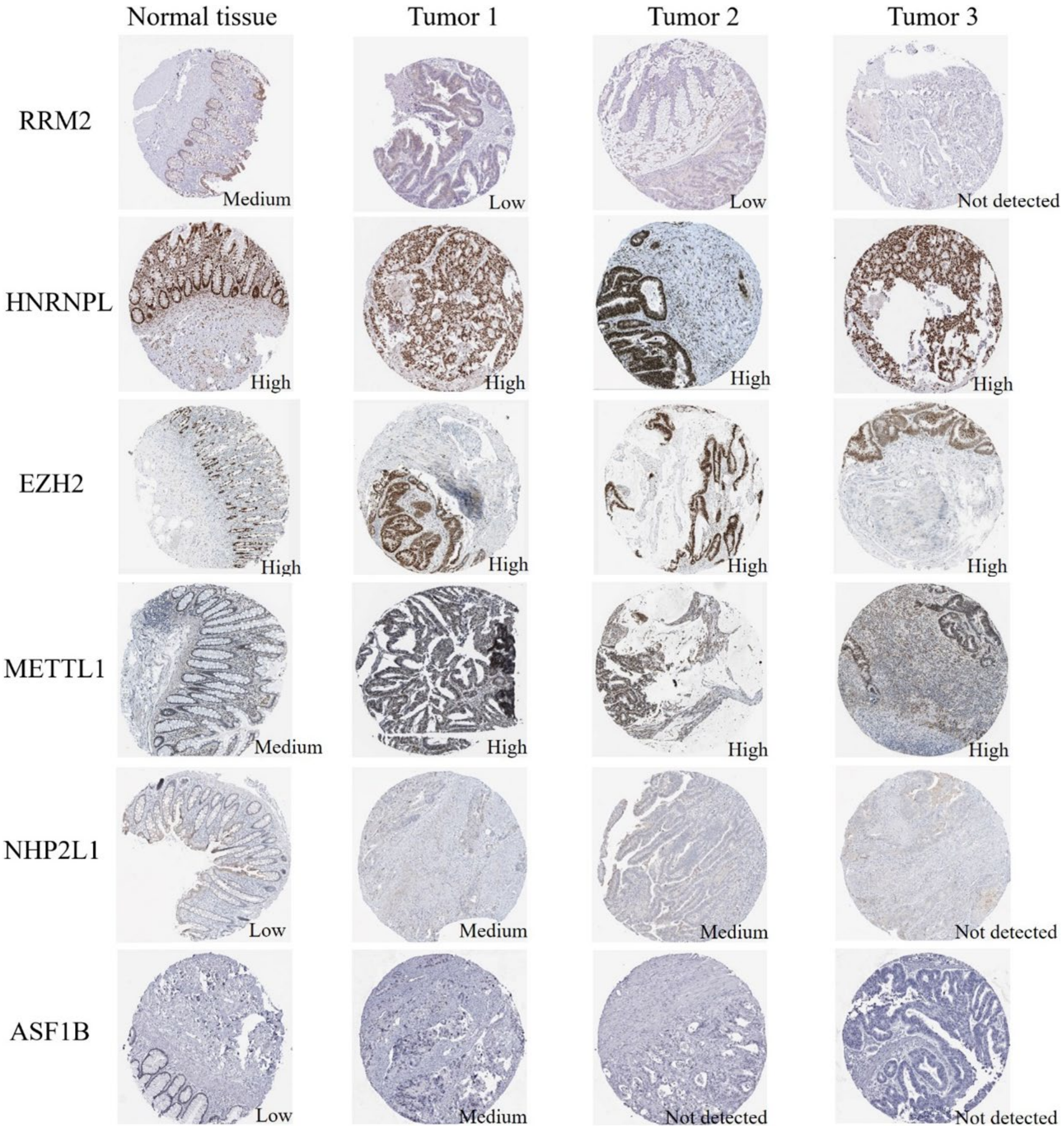

SUPPLEMENTARY FIGURE 5. The protein expression levels of six hub genes (RRM2, HNRNPL, EZH2, METTL1, NHP2L1 and ASF1B) in normal rectum tissues and colorectal cancers detected by immunohistochemistry (IHC) as shown on the Human Protein Atlas (HPA) online database.
